# Spatial effects of environmental regulation on high-quality economic development: From the perspective of industrial upgrading

**DOI:** 10.3389/fpubh.2023.1099887

**Published:** 2023-01-27

**Authors:** Yangyang Li, Weijiang Liu

**Affiliations:** School of Business and Management, Jilin University, Changchun, China

**Keywords:** environmental regulation, industrial structure upgrading, spatial heterogeneity, regional economy high-quality development, sustainable development

## Abstract

Studying the spatiotemporal heterogeneity of environmental regulations on high-quality regional economic development is of enormous practical value in the context of sustainable economic, social, and environmental development. Only a few studies, however, examined the regional heterogeneity of environmental regulation affecting economic development from the standpoint of upgrading the industrial structure. This research investigated the spatial distribution traits of high-quality regional development based on the construction of a comprehensive assessment index system for high-quality economic development. The economic geography-nested spatial Durbin model is then used to perform an empirical test. The findings demonstrate that (1) high-quality economic development has visible spatial heterogeneity, with strong local spatial agglomeration between regions; (2) environmental regulation and the modernization of the industrial structure are significant variables influencing high-quality economic development, but their development is not balanced; and (3) environmental policies promote high-quality regional development through a distinct channel. Formal environmental regulation promotes economic development through rationalizing industrial structure, while informal environmental regulation does so through upgrading the industrial structure. Further, both kinds of environmental regulation have positive spatial spillover effects on adjacent areas. Therefore, the regional heterogeneity of environmental regulation and industrial structure is of great significance in promoting the high-quality and sustainable development of regional economies.

## 1. Introduction

The Chinese economy has experienced extraordinary growth during the last few decades (1). However, over time, the extensive growth model has led to significant energy waste and progressively worse environmental issues. This is incompatible with the new socialist development paradigm and has become a major impediment to China's sustained growth (2–4). The report of the 20th National Congress of the Communist Party of China has clearly stated that China should adhere to the theme of promoting high-quality development, organically combine the implementation of the strategy of expanding domestic demand with deepening supply-side structural reforms, accelerate the establishment of a new development pattern, and promote the effective improvement of quality and reasonable growth of the economy. The upgrading of the industrial structure can deepen the structural reform of the supply side through the spontaneous force of the market and adjust the industrial structure, which is an important force in promoting stable economic growth (5, 6). Environmental regulation (ER) is a crucial instrument for businesses to execute environmental protection measures, as it serves as a critical measure for environmental governance and an institutional guarantee for fostering economic development (7, 8).

Additionally, it is also a key path to coordinate industrial structure upgrading and high-quality economic development (EDQ). Reasonable ER can promote EDQ while promoting industrial modernization and lowering pollutants. However, different ER have various paths for acting on industrial structures, and the effects of solving environmental problems are also different. Therefore, developing effective environmental policies and reversing the conventional dependency on the growth of pollution-intensive firms will help to increase the ability of businesses to regulate pollution and the effectiveness of resource allocation, thereby encouraging sustainable economic and social development in harmony with the environment (9–11). Clarifying the mechanisms of ER, industrial upgrading, and EDQ is a key measure to achieve high-quality economic development against the realistic background of comprehensively deepening reforms and promoting EDQ in China. In addition, this is a crucial step in creating a regional economic structure and land-use system with complementary advantages and high-quality development.

Research on EDQ mainly focuses on three aspects. The first is connotation. Ren Baoping pointed out that high-quality development is the advanced and optimal state of the EDQ (12). Wang Xicheng proposed that the core connotation of EDQ is high-quality, high-efficiency, and high-stability supply systems (13). Gao Peiyong believed that EDQ lies in shifting from focusing on “speed advantage” to pursuing “quality advantage” (14). The second is the evaluation system. Shifu measured the level of EDQ from the three perspectives of development fundamentals, social achievements, and ecological achievements. Ou Jinfeng and Gao Zhigang have combined the five major development concepts in building an index system for EDQ from the five dimensions of innovation, coordination, green, openness, and sharing (15, 16). The third is the realization path. Li Mengxin and Ren Baoping believed that the path chosen for EDQ in the new era was to reconstruct the driving force of technological innovation and deeply embed green productivity (17). Jin Pei proposed that, in the process of achieving EDQ, we should focus on alleviating or even curbing the ecological and environmental problems brought about by economic development (18).

Environmental regulations are environmental standards or actions created by the government to directly or indirectly regulate economic activity and to address the issue of harmful externalities from environmental pollution (19). There are primarily two perspectives in the current research on ER and EDQ. The first view is that the improvement of ER has inhibited enterprises from implementing emission reduction actions. Strict ER leads enterprises to expand output and increase pollution emissions to maximize profits, which is detrimental to the growth of EDQ (20). The second point is that appropriate regulatory measures can encourage enterprises to reduce pollution emissions through green innovation (21, 22), promoting EDQ (23). According to the different types of ER, some scholars divide it into command-and-control, market-type, and informal-type (24), and two types are also sometimes used: formal regulation (FER) and informal regulation (IER) (25). The formal type, which primarily includes the command-and-control and market incentive types (26), belongs to government conduct. It dominates in coordinating environmental protection and economic development (27, 28). The informal type is a non-governmental organization or individual behavior, including public participation and voluntary regulation. When facing higher environmental pollution control needs, IER automatically completes environmental protection agreements through negotiation and so on. It regulates environmental pollution behaviors, achieving higher environmental benefits (29, 30). Different types of ER have varying effects on EDQ due to variations in implementation strategies. Ignoring this difference will lead to deviations in the results of relevant environmental policy evaluations (31, 32). Therefore, we should carefully evaluate how different policies will affect economic development to coordinate ER and EDQ (33).

Industrial upgrading is an important driving force for optimizing resource allocation, promoting kinetic energy conversion, and reducing pollutant emissions. Appropriate ER can encourage enterprises to carry out R&D innovation, optimize resource allocation, and effectively alleviate the contradiction between the economic and ecological systems (34). ER's effects on industrial modernization have always been a vital scholarly concern (35–38), but no unified conclusion has been reached yet. Some scholars believe that ER can promote industrial restructuring (39, 40). Driven by ER, social production materials flow to low-pollution and high-efficiency industries, stimulating high-pollution and low-efficiency enterprises to carry out technological innovation. This process has effectively promoted energy savings and efficiency improvements in enterprises as well as industrial upgrading while reducing emissions (41). For example, Li Hong et al. found that ER can force industrial optimization, thus promoting industrialization's upgrading (42). Kivimaa and Kern discovered that environmental policies encourage technical innovation among businesses, which helps to upgrade the industrial structure (43). Some scholars hold the opposite view, arguing that the cost effect caused by the implementation of ER has inhibited the upgrading of industrial structures (44–46). For example, Liu Jianhua believed that ER had significantly increased the pollution control costs of enterprises, reduced productive investment, and hindered the upgrading and transformation of industrial structures (47). Meng Hao found that ER is not conducive to industrial structure upgrading by constructing the spatial Durbin model (48). It should be emphasized that, while the empirical test for this research produced some results, the conclusions are inconsistent across countries due to variations in environmental laws, sample sizes, and research methodologies. More research needs to be done on ER and modernizing industrial structures.

The relationship between ER, industrial upgrading, and EDQ has been covered in earlier research and has achieved considerable research results, but there are still some deficiencies. First, most of the relevant discussions on promoting EDQ through ER focus on the level of FER while ignoring the contribution of IER. Although some scholars in recent years have proposed that IER is an essential factor affecting EDQ, relevant research has not yet formed a consensus and lacks systematic analysis. Second, the existing literature explores the connection between ER and EDQ from many angles. However, little research was done on the synergistic effects of multiple ERs on EDQ. Most studies are only conducted from one perspective, failing to consider potential mutual limitations across policies. Finally, ER and EDQ, as well as ER and industrial upgrading, are frequently studied in isolation in previous literature, making it hard to reflect on the relationship between them. Few studies link these three items to explore their relationship and spatial spillover effects.

The following are the primary contributions of the article in comparison to previous research: First, this study quantitatively analyzes the comprehensive evaluation index system of EDQ and its spatial distribution and further studies the systemic effect of ER from the perspective of industrial upgrading on this basis. This can provide more targeted suggestions for formulating China's macroeconomic and environmental policies. Second, the article divides environmental regulations into formal and informal environmental regulations to examine their independent and interactive effects on EDQ. Third, this article puts dual ER, industrial upgrading, and EDQ in the same research framework and explores the relationship between them and their temporal and spatial characteristics. Therefore, it can provide a reference for promoting environmental governance, facilitating the change of industrial structure, and realizing coordinated EDQ. Fourth, this study discusses how the industrial transformation paths of FER and IER affect EDQ and provides a valuable reference for formulating and implementing ER policies in the future. Finally, this study divides regions according to spatial geographic location for heterogeneity and replaces the spatial weight matrix for robustness testing, which provides an empirical basis for policymakers to improve the quality of economic development.

The remainder of the essay is organized in the following manner. The theoretical analysis and research assumptions are covered in theoretical analysis and research assumptions section. The resources and procedures are described in materials and methods section. Results and discussion section contains the empirical findings as well as the analysis. Robustness and endogeneity test section is the robustness test and the endogeneity test. The conclusions and their consequences for policy are provided in conclusions and policy implications section.

## 2. Theoretical analysis and research assumptions

Due to the influence of numerous elements, including regulation type, regulation intensity, and policy execution, the effect of ER on EDQ is ambiguous (49, 50). This influence is primarily accomplished by combining the “cost offset effect” and the “innovation compensation effect” as the transmission method. On the one hand, increased environmental legislation has forced businesses to pay a premium to reduce pollution. Enterprise innovation's cost effect cannot now be balanced out by its compensation effect (51, 52). In particular, the increased cost of environmental pollution control will reduce the initial investment in the productive output of businesses, skew resource allocation to businesses, impede the development of green technologies, and subtly impede EDQ. As opposed to this, when the innovation compensation impact outweighs the cost offset effect, the company will reduce pollution emissions through the invention of green technology, increase its production efficiency, and balance the cost effect, occupying a favorable position in the market competition and driving EDQ. The effect of ER on the EDQ is thus ambiguous. ER is divided into two categories in this study: FER and IER. Compared to IER, the cost-effectiveness of FER is more visible, but the innovation compensation effect is less clear and even has a negative impact. FER describes the restrictions put in place by the government to force polluting companies to reduce emissions through administrative action and market incentives. Therefore, polluting businesses will incur a disproportionately high cost when they succeed in maximizing profits. Due to the informal and non-mandatory nature of IER, its influence on resource allocation distortion is frequently subtle. Therefore, it will not have a significant effect on business expenses. More businesses will embrace an environmentally friendly company philosophy as the idea of green and sustainable development becomes more widely accepted in society. Companies will increase their social impact and brand awareness through this technique. Thus, informal environmental control will effectively encourage the “innovation compensation” effect of businesses and realize the “win-win” of environmental protection and productivity improvements. The following hypotheses are put forth in this study based on the analysis shown above:

H1: Varying ER has different degrees of impact on EDQ; FER has a negative effect, and IER has a positive effect.

Industrial upgrading is the reallocation of production factors among distinct economic sectors and industries (53), that is, the process of promoting industrial structure rationalization (TR) and advanced development (TS). Industrial upgrading can encourage the coordinated growth of different industries within the national economy, enabling those industries to flourish in ways compatible with EDQ. The TR reflects the degree of coordination among industries and the effective use of resources. It gauges how closely each region's factor input structure and industrial structure are coupled (54). By optimizing the proportion of the industrial structure, the TR adjustment can accomplish complete resource protection, lower energy consumption, minimize pollutant emissions, and foster EDQ. Factors of production, including labor force, capital, and natural resources, move freely between industries due to the shift in the force that drives economic development. The industrial structure is constantly shifting from the secondary to the tertiary industry, thereby driving the structural adjustment of various production factors (55–57). TS describes the process of changing the regional industrial structure from a low-level structure dominated by labor-intensive industries to a high-level structure dominated by technology-intensive industries. The advanced industrial system promotes the transfer of factor resources from high-consumption to low-consumption industries, which helps store energy and reduce pollution. At the same time, the continuous development of knowledge-intensive industries can drive technological progress, thereby driving the upgrading of traditional production methods and the use of advanced production methods. This is conducive to achieving EDQ. On the basis of the analysis above, the following premises are advanced:

H2: TR and TS can both promote the regional EDQ.

Environmental regulation has a mandatory restraint force on an enterprise's production and pollution discharge behavior and intends to safeguard the environment. Microscopically, it can encourage companies to implement pollution control and green innovation. Macroscopically, it can direct the conversion of polluting businesses into clean ones and realize an important step from the secondary industry to the tertiary industry. ER affects the direction of industrial structure transformation through various means, affecting EDQ (32). FER controls pollution emissions at the source by clarifying each market entity's pollution reduction and corporate responsibilities, thereby affecting EDQ. This impact can be divided into two aspects. First, the “environmental compliance costs” of different types of industries are different. Pollution-intensive industries need to bear higher environmental costs and have weak technical research capabilities, making it difficult to offset costs through innovation effects. Clean industries have green competitive advantages and tend to increase R&D investment to cope with rising environmental costs. Strict ER leads to the reconfiguration of market shares, which promotes the continuous improvement of the level of coordination among various industries, thus promoting the process of rationalizing industrial structure. Second, moderate ER will stimulate businesses to innovate. Driven by technological progress, low-productivity sectors have gradually withdrawn from the market, and high-productivity sectors have continued refining their labor division. This will drive the transformation of the industrial structure to an advanced level, move the frontier of social production forward, and promote EDQ. IER refers to the spontaneous completion of environmental protection agreements by the public, the media, or other environmental protection organizations through negotiations and consultations to standardize environmental pollution control behaviors when faced with higher environmental pollution control needs. The incentive effect of IER on industrial structure adjustment is mainly reflected in two aspects. First, IER can directly exert pressure on polluting industries by acting on productive demand, prompting enterprises to reform the industrial structure (58) and advance the regional EDQ. The second is that the demand for environmentally friendly goods and services is growing along with public awareness of environmental protection. These factors encourage enterprises to increase research and application of green technologies, which helps to upgrade the industrial structure and raise the standard of EDQ. The following assumptions are therefore outlined in this paper:

H3: Dual ER can effectively force industrial transformation and upgrading, promoting EDQ.

Environmental regulation policies are multifield and complex, so it is unreasonable to only emphasize the impact of a single policy (59). Ignoring the heterogeneity of ER might result in deviations throughout policy evaluation results that are not favorable for regional EDQ. Therefore, coordinating multiple policies has become an inevitable choice to encourage EDQ. Specifically, one is that local governments can still not eliminate the influence of the GDP-only theory. They typically make a trade-off between ER and economic performance. At this time, informal environmental regulation organizations such as the public, media, and environmental protection associations are required to supervise the implementation of FER. The strength of IER is used to supervise the effective implementation of FER. Therefore, dual ER work together to promote EDQ. The other is that the promulgation of FER represents the government's determination to control environmental pollution. Its extensive influence in society is conducive to raising the public's awareness of environmental protection and enhancing social groups' understanding of pollution control. That is, FER can encourage the formation of IER, which will then support EDQ under the influence of consumption and industrial structure adjustment effects. Thus, FER and IER can interact with each other to promote EDQ. In light of the previous analysis, the following assumptions are proposed in this paper:

H4: The interaction between FER and IER promotes regional EDQ.

## 3. Materials and methods

### 3.1. Design of research

The empirical analysis presented in this research uses the Spatial Durbin Model (SDM) to investigate the effects of ER and industrial upgrading on EDQ and its spatial impact. This study uses the two components of industrialization advancement (TS) and rationalization (TR) to measure industrial upgrading. Based on this measurement, the interaction between ER and industrial transformation and modernization is introduced. The specific model settings are as follows:


(1) $$\begin{array}{l} \begin{array}{l} \ln\;EDQ_{it}=\rho W_{ij}\ln\;EDQ_{it}+\beta_{1}\ln\;FER_{it}+\beta_{2}\ln\;TS_{it}+\beta_{3}\ln\;TR_{it}\\ \;+\beta_{4}\ln\;FER_{it}\times TS_{it}+\beta_{5}\ln\;FER_{it}\times TR_{it}\;+\beta_{6}X_{it}\\ \;+\delta_{1}W_{ij}\ln\;FER_{it}+\delta_{2}W_{ij}\ln\;TS_{it}+\delta_{3}W_{ij}\ln\;TR_{it}\\ \;+\delta_{4}W_{ij}\ln\;FER_{it}\times TS_{it}+\delta_{5}W_{ij}\ln\;FER_{it}\times TR_{it}\\ \;+\delta_{6}W_{ij}X_{it}+\mu_{i}+\gamma_{t}+\varepsilon_{it} \end{array} \end{array}$$

(2) $$\begin{array}{l} \begin{array}{l} \ln\;EDQ_{it}=\rho W_{ij}\ln\;EDQ_{it}+\beta_{1}\ln\;IER_{it}+\beta_{2}\ln\;TS_{it}+\beta_{3}\ln\;TR_{it}\\ \;+\beta_{4}\ln\;IER_{it}\times TS_{it}+\beta_{5}\ln\;IER_{it}\times TR_{it}+\beta_{6}X_{it}\\ \;+\delta_{1}W_{ij}\ln\;IER_{it}+\delta_{2}W_{ij}\ln\;TS_{it}+\delta_{3}W_{ij}\ln\;TR_{it}\\ \;+\delta_{4}W_{ij}\ln\;IER_{it}\times TS_{it}+\delta_{5}W_{ij}\ln\;IER_{it}\times TR_{it}\\ \;+\delta_{6}W_{ij}X_{it}+\mu_{i}+\gamma_{t}+\varepsilon_{it} \end{array} \end{array}$$

Equations (1) and (2) represent the spatial panel Durbin model of formal and informal environmental regulation, respectively. Among them, ρ represents the spatial correlation coefficient of the dependent variable; i demonstrates the region; t shows time; W_ij_ reflects the geographic economic nesting spatial weight matrix; EDQ_it_ represents high-quality economic development; FER_it_ and IER_it_ represent formal and informal environmental regulations, respectively; TS_it_ and TR_it_ represent the industrial structure's advancement and rationalization, respectively; and Xit displays the control variable array. The space and time control variables are symbolized by μ_i_ and γ_i_, including both. ε_it_ denotes the random error term.

Researching the combined regulatory impact of FER and IER is necessary to be more likely with the realistic background. Therefore, the interaction terms FER and IER are introduced into the aforementioned spatial Durbin model. Following is the model for such a corresponding panel expansion:


(3) $$\begin{array}{l} \begin{array}{l} \ln\;EDQ_{it}=\rho W_{ij}\ln\;EDQ_{it}+\beta_{1}\ln\;FER_{it}+\beta_{2}\ln\;IER_{it}+\beta_{3}\ln\;FER_{it}\\ \;\times \ln\;INER_{it}+\beta_{4}\ln\;TS_{it}+\beta_{5}\ln\;TR_{it}+\beta_{6}\ln\;FER_{it}\times TS_{it}\\ \;+\beta_{7}\ln\;FER_{it}\times TR_{it}+\beta_{8}\ln\;IER_{it}\times TS_{it}+\beta_{9}\ln\;IER_{it}\\ \;\times TR_{it}+\beta_{10}X_{it}+\delta_{1}W_{ij}\ln\;FER_{it}+\delta_{2}W_{ij}\ln\;IER_{it}\\ \;+\delta_{3}W_{ij}\ln\;FER_{it}\times \ln\;IER_{it}+\delta_{4}W_{ij}\ln\;TS_{it}+\delta_{5}W_{ij}\ln\;TR_{it}\\ \;+\delta_{6}W_{ij}\ln\;FER_{it}\times TS_{it}+\delta_{7}W_{ij}\ln\;FER_{it}\\ \;\times TR_{it}+\delta_{8}W_{ij}\ln\;IER_{it}\times TS_{it}+\delta_{9}W_{ij}\ln\;IER_{it}\times TR_{it}\\ \;+\delta_{10}W_{ij}X_{it}+\mu_{i}+\gamma_{t}+\varepsilon_{it} \end{array} \end{array}$$

Among them, the meanings of the symbols are the same as in the above formulas (1) and (2).

### 3.2. Variable choice

#### 3.2.1. Explained variable

The explained variable is high-quality economic development (EDQ). EDQ is a shift in the economic growth model, moving from high-speed growth's quantitative change to its qualitative change. This improvement makes the economy run more effectively, the industrial structure more sensible, the services and goods better, the economy healthier and more sustainable, the society more egalitarian and harmonious, and the ecosystem greener (12). EDQ stands for high-level economic development. The degree of EDQ can only be effectively quantified by building an efficient method for measuring it (13). The majority of the current literature is based on the five development concepts of innovation, coordination, greenness, openness, and sharing to create a high-quality development index evaluation system. However, the EDQ index system should not only reflect the current condition of economic development but also pay attention to the operating status of economic development (60). The indicator system created by the five development concepts may fail to pay attention to the operational efficiency of economic development, and it will invariably have issues such as complex indicators that result in poor indicator selection efficiency (61). This study builds an index system from six dimensions: economic efficiency, economic structure, economic stability, innovation level, green environmental protection, and public welfare, combining existing research and integrating it with the concept of EDQ (15–18, 62). The specific indicators are shown in Table 1, and index weights are calculated using the entropy method.

**Table 1:** Comprehensive indicator system for high-quality economic development.

**Primary indicators**	**Secondary indicators**	**Calculation method**	**Attributes**
Economic efficiency	Labor efficiency	GDP/number of employees	Positive
	Land efficiency	Grain production/sown area of grain crops	Positive
	Capital efficiency	GDP/social fixed asset investment	Positive
	Energy efficiency	GDP/10,000 tons of standard coal	Positive
	Full factors production rate	Full factors production rate	Positive
Economic structure	Advanced industrial structure	Advanced index	Positive
	Rationalization of industrial structure	Rationalization index	Negative
	Urbanization rate	Urban population/total population	Positive
	Consumption rate	Consumption expenditure/GDP	Positive
	Binary gamma	Binary gamma	Negative
	Binary contrast coefficient	Binary contrast coefficient	Positive
	Investment rate	Gross capital formation/GDP	Positive
	Deposit balance/GDP	Deposit balance/GDP	Positive
	Loan balance/GDP	Loan balance/GDP	Positive
	Foreign trade openness	Total import and export trade/GDP	Positive
	Openness to foreign investment	Outward direct investment/GDP	Positive
Economic stability	Consumption index	Consumer price index	Negative
	Economic growth rate	Regional real economic growth rate	Negative
	Production index	Producer price index	Negative
	Unemployment rate	Urban registered unemployment rate	Negative
	Retail index	Commodity retail price index	Negative
Innovation level	Technological innovation output	The number of granted patent applications	Positive
	R&D investment intensity	Internal expenditure of R&D funds/GDP	Positive
	Technical turnover	Technology market turnover/GDP	Positive
	Technology investment intensity	Science-technology expenditure/financial expenditure	Positive
	Market index	Market index	Positive
Green development	Energy consumption	Total energy consumption/GDP	Negative
	Exhaust emissions	Total SO_2_ emissions/GDP	Negative
	Wastewater discharge	Total industrial wastewater discharge/GDP	Negative
	Power consumption	Electricity consumption/GDP	Negative
	Solid waste discharge	Industrial solid waste generation/GDP	Negative
	Intensity of environmental governance	Environmental protection expenditure/financial expenditure	Positive
	Green area	Green coverage	Positive
Public welfare	GDP per capita	GDP per capita	Positive
	Workers' Compensation Proportion	Workers' compensation/real GDP	Positive
	Population mortality	Population mortality	Negative
	Years of education per capita	Average years of education for people	Positive
	Per capita education expenditure	Education expenditure/total population	Positive
	Health technicians per 1,000 population	Number of health technicians/total resident population*1,000	Positive
	Old-age insurance coverage	Number of people participating in basic pension insurance/total population	Positive
	Health insurance coverage	Urban medical insurance participants/total urban population	Positive

#### 3.2.2. Independent variables

Formal environmental regulation (FER): This study selects the completed investment in industrial pollution control to measure the intensity of FER. This investment in business environmental protection reflects the government's attention to environmental pollution and its determination to control it, which is suitable for FER.

Informal environmental regulation (IER): In accordance with the measurement method by Pargal and Wheeler (63), the indicators of economic status, population size, educational attainment, and age distribution are selected to be measured by the entropy method, representing IER.

Industrial upgrading includes rationalizing industrial structure (TR) and advancing industrial structure (TS).

Rationalizing industrial structure (TR): With reference to the study by Cheng et al. (64), we assessed TR using the inverse of the Theil index. The Theil index maintains the theoretical basis and economic meaning of the degree of structural deviation while also considering the relative significance of sectors and avoiding absolute value computation. The specific formula is as follows:


(4) $$\begin{array}{l} TR=1/\overset{n}{\underset{i=1}{\sum }}\left(\frac{Y_{i}}{Y}\right)\ln\;\left(\frac{Y_{i}}{L_{i}}/\frac{Y}{L}\right) \end{array}$$

Here, Y represents the gross regional product. L symbolizes the employed population. Yi/Y symbolizes the employed population as a symbol for the output structure. Y/L represents productivity. The industrial structure is more logical and sensible as the TR increases and is more out of equilibrium as the TR decreases.

Advanced industrial structure (TS): TS reflects the service-oriented tendency of the economic structure and the transformation process of the industrial structure from a lower-level form to a higher-level form. Therefore, the ratio of secondary sector production quality to the tertiary sector is measured in this study.

Control variables: This research includes control factors to decrease the forecast error caused by omitted regressors and to provide an objective evaluation of the policy effect. Specifically, it considers economic development (Vgdp), which is measured by per capita GDP; marketization (Vmark), which is expressed by the total marketization index proposed in the “China Marketization Index”; financial development level (Vfin) is represented by the balance of various deposits of financial institutions as a percentage of the regional GDP; and technological innovation (Vrd), the ratio of R&D expenditure to GDP, is known as the technological innovation metric.

### 3.3. Data sources

We used the provincial panel data from 30 provinces (municipalities, autonomous regions) in China from 2002 to 2020 (excluding Hong Kong, Macau, Taiwan, and Tibet) for empirical analysis. The primary sources are the “China Statistical Yearbook,” “China Financial Yearbook,” “China Environmental Yearbook,” and the EPS database. Some missing data were provided by linear interpolation. The variables, as mentioned earlier, are taken logarithmically to eliminate possible heteroskedasticity issues in the data. Table 2 displays the summary statistics for the variables.

**Table 2:** Variables' descriptive statistics.

**Variables**	**Mean**	**Std.Dev**	**Min**	**Max**	**Observations**
lnEDQ	−1.650	0.390	−2.380	−0.280	570
lnFER	2.400	1.140	−3.040	4.950	570
lnIER	−1.760	0.430	−2.670	−0.130	570
lnTS	−0.040	0.390	−0.700	1.670	570
lnTR	1.740	0.820	0.130	4.840	570
lnVgdp	1.090	0.790	−1.120	2.800	570
lnVmark	1.830	0.320	0.850	2.480	570
lnVfin	0.440	0.330	−0.290	1.720	570
lnVrd	−4.480	0.670	−6.350	−2.740	570

## 4. Results and discussion

### 4.1. Calculation results of high-quality economic development

**Figure 1:**
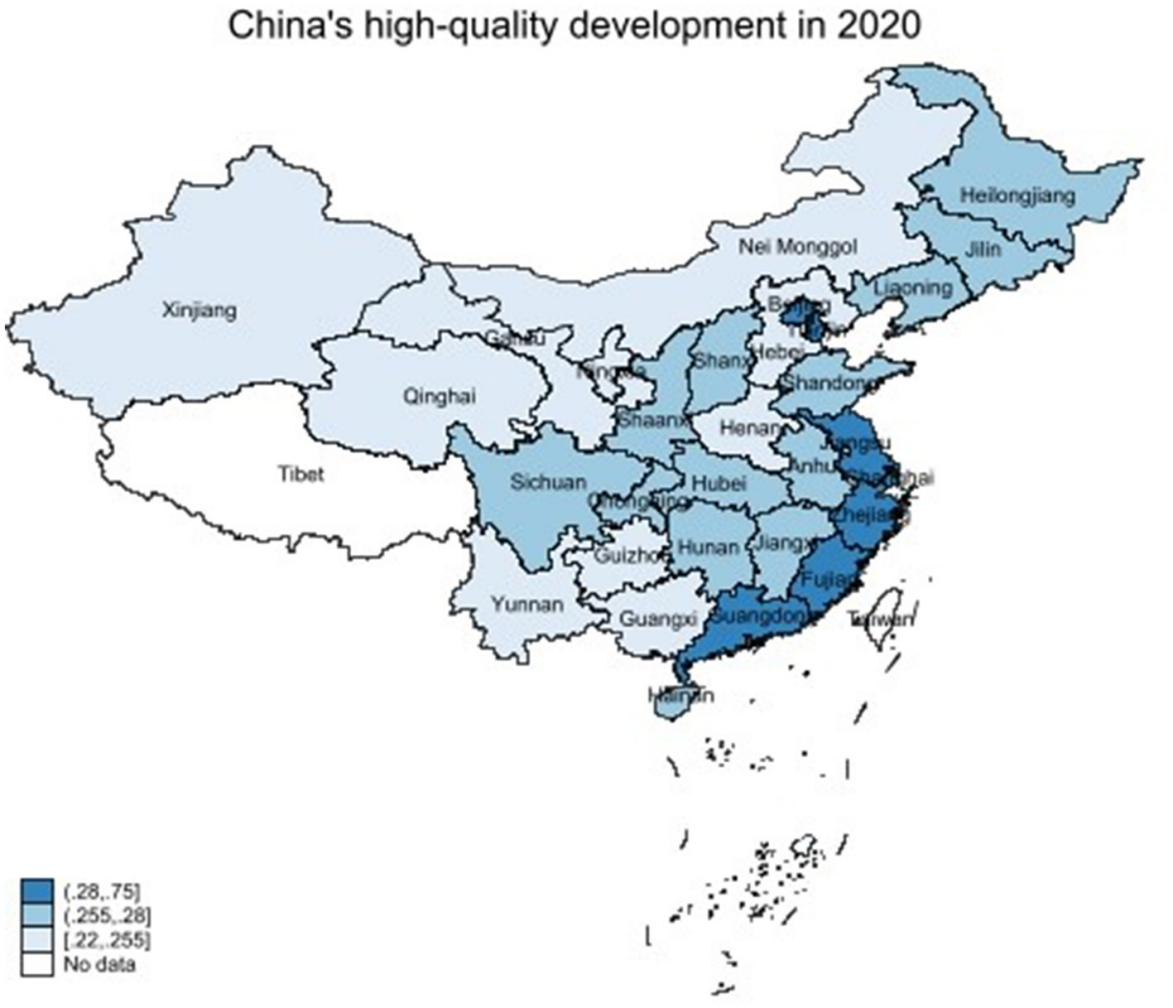
Regional distribution of high-quality development.

The entropy method was employed to calculate the degree of EDQ in 30 Chinese provinces in accordance with the research methods discussed above. To more intuitively show the spatial correlation between EDQ in various regions, this study draws a spatial distribution map of EDQ. Figure 1 shows the regional distribution of EDQ at the provincial level in 2020. Economic development is divided into three levels. The darker the color, the higher the level of EDQ. From the perspective of space, the high-value agglomeration areas for EDQ are mainly coastal, economically developed areas. In contrast, the northeast, northwest, and underdeveloped areas in the interior are primarily low-value agglomerations. This shows that the EDQ between regions has a strong local spatial agglomeration effect: high–high (H–H) or low–low (L–L) agglomeration.

### 4.2. Spatial correlation test

#### 4.2.1. Moran's index test

This study used the exploratory spatial data analysis method to test the global spatial correlation of the EDQ level of each province according to the global Moran's index (GMI). Considering the combined effect of economic and geographical factors, this paper employs that spatial matrix of geographic socioeconomic nesting to examine regional EDQ's spatial autocorrelation. Table 3 displays the test results.

**Table 3:** Moran's index testing.

**Year**	**GMI**	**Z**	**Year**	**GMI**	**Z**
2002	0.524^***^	5.359	2012	0.506^***^	5.328
2003	0.481^***^	4.934	2013	0.514^***^	5.420
2004	0.503^***^	5.156	2014	0.505^***^	5.348
2005	0.538^***^	5.533	2015	0.500^***^	5.344
2006	0.534^***^	5.490	2016	0.480^***^	5.183
2007	0.529^***^	5.457	2017	0.487^***^	5.375
2008	0.527^***^	5.480	2018	0.466^***^	5.158
2009	0.523^***^	5.494	2019	0.474^***^	5.198
2010	0.524^***^	5.510	2020	0.469^***^	5.126
2011	0.516^***^	5.415			

^***^, ^**^, ^*^represent the 1, 5, and 10% levels of significance, respectively.

The EDQ index's GMI values all passed the test at a significance level of 1% and were significantly positive. This demonstrates that EDQ is significantly spatially correlated positively. The EDQ index has an apparent spatial agglomeration effect among regions. High (low) adjacent interprovincial units are relatively agglomerated, showing a relatively spatial solid agglomeration pattern.

**Figure 2:**
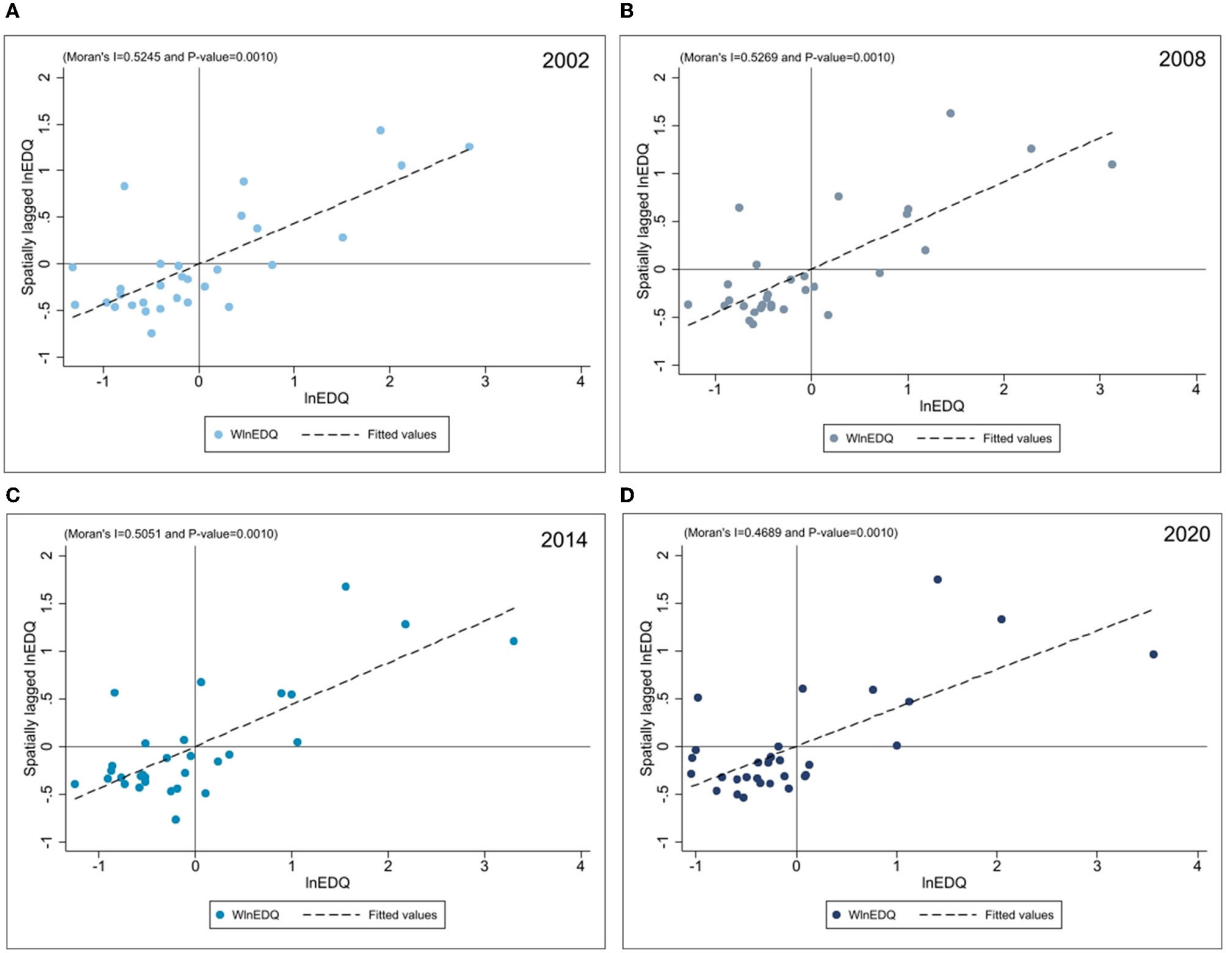
**(A–D)** Local Moran's index scatters plot.

This study draws a partial Moran index scatter plot of EDQ for 2002, 2008, 2014, and 2020, depicted in Figures 2A–D, to investigate further if there are variations in the spatial correlation of EDQ in various regions. The EDQ Moran scatter plot shows that most provinces are clustered among the first and third quadrants, presenting the characteristics of “H–H” or “L–L” agglomeration. This demonstrates that EDQ has an apparent spatial agglomeration tendency. Specifically, comparing those figures in 2002 and 2020, we can see that the percentage in regions with “L–L” agglomerations expanded significantly. The first and third quadrants included 24, 25, 24, and 25 provinces in 2002, 2008, 2014, and 2020, respectively. The spatial distribution of EDQ is mainly characterized by high-high or low-low aggregation, which means that provinces with high quality are usually adjacent to other regions with high-quality economic development, and vice versa.

#### 4.2.2. Spatial model selection test

There are three common spatial econometric models, namely the spatial lag model (SAR), the spatial error model (SEM), and the spatial Durbin model (SDM). The analysis model was selected according to the spatial econometric model selection method proposed by Elhorst (65), and the results are shown in Table 4. It is appropriate to choose SDM among SAR, SEM, and SDM by the LM test. The Hausman test results are significant at the 1% level, so this study chooses the fixed effect model. The Wald and LR tests rejected the hypothesis that the SDM model can be simplified to SAR and SEM. Therefore, the fixed-effect spatial Durbin model is this study's optimal spatial econometric model.

**Table 4:** Spatial model correlation test results.

**Test**	**Type**	**Statistics**	**Results**
LM test	LM test (error)	130.611^***^	SDM model
	Robust LM test (error)	14.924^***^	
	LM test (lag)	185.069^***^	
	Robust LM test (lag)	69.382^***^	
Hausman test	Difference in coeffs not systematic	251.77^***^	Fixed effects
Wald test	SAR simplifies to SDM	92.02^***^	Refuse to simplify
	SEM simplifies SDM	48.14^***^	
LR test	SAR nested within SDM	85.44^***^	Refuse to simplify
	SEM nested within SDM	50.42^***^	

^***^, ^**^, ^*^represent the 1, 5, and 10% levels of significance, respectively.

### 4.3. Empirical results

This study built a spatial Durbin model in accordance with the previous test to investigate the implications of dual ER and industrial upgrading on EDQ. Table 5 presents the outcomes. The model's capacity to fit data well and the reliability of the regression findings are both demonstrated by the sigma2_e and R^2^ statistics. The spatial lag term's coefficients ρ are all significantly positive, which confirms that the local EDQ has a positive externality to the surrounding areas. It shows that the influence of spatial externalities between regions should not be ignored when studying the regional EDQ.

**Table 5:** Estimation results.

**Variables**	**Model 1**	**Model 2**	**Model 3**
lnFER	−0.0081^**^		0.0327^*^
	(−2.05)		(1.96)
lnIER		0.144^***^	0.0809^**^
		(4.07)	(2.06)
lnFIER			0.0208^**^
			(2.34)
lnTS	0.0710^***^	0.0336^*^	0.0397^**^
	(4.35)	(1.94)	(2.27)
lnTR	0.0189^**^	0.0371^***^	0.0383^***^
	(2.20)	(3.87)	(3.93)
lnFERTS	−0.0160^**^		−0.0285^***^
	(−2.17)		(−3.59)
lnFERTR	0.0126^***^		0.0136^**^
	(3.03)		(2.37)
lnIERTS		0.0474^**^	0.0572^***^
		(2.21)	(2.59)
lnIERTR		−0.0278^**^	−0.0677^***^
		(−1.98)	(−4.97)
lnVgdp	0.195^***^	0.0533^***^	0.105^***^
	(8.45)	(5.08)	(8.12)
lnVmark	0.183^***^	0.113^***^	0.142^***^
	(6.27)	(6.39)	(7.86)
lnVfin2	0.138^***^	0.0583^**^	0.0904^***^
	(5.43)	(2.32)	(3.66)
lnVrd	0.0507^***^	0.0573^***^	0.0587^***^
	(3.85)	(4.25)	(4.37)
ρ	0.584^***^	0.527^***^	0.481^***^
	(17.21)	(13.51)	(11.78)
sigma2_e	0.00221^***^	0.00235^***^	0.00221^***^
	(16.63)	(16.64)	(16.67)
R^2^	0.933	0.887	0.903
N	570	570	570

lnFIER represents lnFER × lnIER, lnFERTS represents lnFER × lnTS, lnFERTR represents lnFER × lnTR, lnIERTS represents lnIER × lnTS, lnIERTR represents lnIER × lnTR. ^***^, ^**^, ^*^represent the 1, 5, and 10% levels of significance, respectively. *t* statistics in parentheses.

Models 1 and 2's regression findings demonstrate that FER's coefficient is significantly negative (−0.0081), which reveals that FER inhibits EDQ. The reason may be that FER policies are easily affected by factors such as government implementation efficiency. The implementation of regulations is not targeted, and the same standards are adopted for the pollution control of different enterprises, which negatively affects the level of EDQ. Currently, “follow the cost effect” dominates, which is consistent with Sinn's findings. The IER's coefficient is significantly positive (0.144), showing that the IER enhances the EDQ, which would align with Michael et al.'s results. The effect of IFE's resource allocation distortion is frequently not evident because of its non-mandatory nature. It will effectively encourage enterprises' innovative compensation effects to achieve a win-win situation of environmental protection and efficiency improvement. The results above demonstrate how different ER forms have distinct effects on EDQ. Hypothesis 1 is verified.

The coefficients of the TS (0.071 in model 1, 0.0336 in model 2) and TR (0.0189 in model 1, 0.0371 in model 2) are both significantly positive, indicating that industry upgrading would promote EDQ. This may be because rational adjustments have promoted the matching of production factor input and output structures, and improved the efficiency of resource allocation, thereby promoting EDQ. The advanced adjustment causes both organizational productivity and service in sector proportion to keep rising. This reduces energy consumption and, as a result, advanced manufacturing pollution, promoting the EDQ even further. That confirms Hypothesis 2.

The interaction term between FER and TR is significantly positive at the 1% level (0.0126). The interaction term between IER and TS is significantly positive at the 5% level (0.0474). The above conclusions indicate that TR has a significant promoting effect on the relationship between FER and EDQ, and TS has a major promoting effect on the relationship between IER and EDQ. ER can promote EDQ by promoting industrial upgrading. The benign interaction between ER and industrial upgrading will help enterprises optimize resource allocation to the greatest extent and promote the continuous improvement of the coordination level among various industries. However, this kind of benign interaction can encourage enterprises to carry out technological transformation and innovation, enhance their pollution treatment capabilities, and thereby support EDQ. Hypothesis 3 is verified.

Model 3 adds the interaction terms of FER and IER in accordance with Models 1 and 2. The regression analysis found that the interaction term's coefficient appeared positively significant. The FER's coefficient changed from to 0.0081 in model 1 to 0.0327 in model 3. The impact of FER on EDQ switched from inhibiting to promoting. FER and IER have a substitution relationship. When FER is weak or ineffective, IER can fully utilize the public and environmental protective groups' oversight strengths to promote EDQ further. IER can well supervise the implementation of FER, and FER can promote the formation of IER. The mechanisms of dual ER complement and encourage each other. Hypothesis 4 is verified.

From the perspective of control variables, the coefficients of economic development level, marketization level, financial development level, and technological innovation level are strongly positive. The enhancement of people's awareness of environmental protection, the improvement of marketization level, the progress of financial development level, and the increase of enterprise R&D investment are all conducive to EDQ moving toward a goal of superb quality.

### 4.4. Space effect decomposition discussion

The coefficients of the spatial Durbin model cannot accurately describe the marginal impact of explanatory variables, and only the decomposed direct effects, indirect effects, and total effects can truly reflect the “local-adjacent” effect (66). The direct implications represent the impact of locally relevant factors on the region's EDQ. The indirect effect is the spillover effect. It reflects the influence of neighboring provinces on the economic development level of the province. It also reflects that neighboring provinces have an impact on their own economic development level and further affect the province's economic development level through a circular feedback system. The total effect is the sum of the direct and indirect effects. The specific calculation process is as follows:

The general form of the spatial Durbin model:


(5) $$\begin{array}{l} Y=\alpha l_{n}+\rho WY+\beta X+\gamma WX+\varepsilon \end{array}$$

It can be simplified into the following structure *via* conversion:


(6) $$\begin{array}{l} \left(I_{n}-\rho W\right)Y=\alpha l_{n}+\beta X+\gamma WX+\varepsilon \end{array}$$

Let L(W) = (I_n_-ρW)^−1^ and S_m_(W) = L(W) (I_n_β_m_ + γ_m_W), then the formula (6) can be written as:


(7) $$\begin{array}{l} Y=\overset{k}{\underset{m=1}{\sum }}S_{m}\left(W\right)X_{m}+L\left(W\right)\alpha l_{n}+L\left(W\right)\varepsilon \end{array}$$

The matrix below is formed through transformation:


(8) $$\begin{array}{l} \left[\begin{array}{c} Y_{1}\\ Y_{2}\\ \vdots \\ Y_{n} \end{array}\right]=\overset{k}{\underset{m=1}{\sum }}\left[\begin{array}{cccc} S_{m}{\left(W\right)}_{11} & S_{m}{\left(W\right)}_{12} & \cdots & S_{m}{\left(W\right)}_{1n}\\ S_{m}{\left(W\right)}_{21} & S_{m}{\left(W\right)}_{22} & \cdots & S_{m}{\left(W\right)}_{2n}\\ \vdots & \vdots & \ddots & \vdots \\ S_{m}{\left(W\right)}_{n1} & S_{m}{\left(W\right)}_{n2} & \cdots & S_{m}{\left(W\right)}_{nn} \end{array}\right]\left[\begin{array}{c} X_{1m}\\ X_{2m}\\ \vdots \\ X_{nm} \end{array}\right]\\ \;+L\left(W\right)\alpha l_{n}+L\left(W\right)\varepsilon \end{array}$$

The first matrix on the right side of the equal sign is the partial differential matrix, and the elements on its diagonal reflect the direct effects, direct = dY_i_/ dX_im_ = S_m_(W)_ii_. Off-diagonal elements reflect spatial spillover, indirect = dY_i_/ DX_jm_ = S_m_(W)_ij_. This part analyzes the decomposition of spatial effects under the same framework that incorporates dual ER and industrial upgrading. Table 6 displays the outcomes.

**Table 6:** Spatial effects decomposition results.

**Variables**	**Direct effect**	**Indirect effect**	**Total effect**
lnFER	0.0319^*^	−0.0218	0.0101
	(1.77)	(−1.17)	(0.29)
lnIER	0.0832^**^	0.0655^**^	0.149^**^
	(2.05)	(2.09)	(2.09)
lnFIER	0.0225^**^	0.0182^**^	0.0407^**^
	(2.36)	(2.20)	(2.32)
lnTS	0.0416^**^	0.0331^**^	0.0747^**^
	(2.34)	(2.33)	(2.38)
lnTR	0.0343^***^	−0.0630^**^	−0.0287
	(3.57)	(−2.07)	(−0.85)
lnFERTS	−0.0295^***^	−0.0238^***^	−0.0533^***^
	(−3.54)	(−3.03)	(−3.39)
lnFERTR	0.0139^**^	0.0112^**^	0.0250^**^
	(2.24)	(2.12)	(2.22)
lnIERTS	0.0597^***^	0.0484^**^	0.108^**^
	(2.58)	(2.29)	(2.49)
lnIERTR	−0.0703^***^	−0.0567^***^	−0.127^***^
	(−4.92)	(−3.92)	(−4.65)
lnVgdp	0.111^***^	0.0890^***^	0.200^***^
	(8.88)	(7.24)	(9.93)
lnVmark	0.150^***^	0.120^***^	0.270^***^
	(8.21)	(5.76)	(7.84)
lnVfin2	0.0972^***^	0.0776^***^	0.175^***^
	(3.62)	(3.56)	(3.73)
lnVrd	0.0608^***^	0.0490^***^	0.110^***^
	(4.42)	(3.75)	(4.28)

lnFIER represents lnFER × lnIER, lnFERTS represents lnFER × lnTS, lnFERTR represents lnFER × lnTR, lnIERTS represents lnIER × lnTS, lnIERTR represents lnIER × lnTR. ^***^, ^**^, ^*^represent the 1, 5, and 10% levels of significance, respectively. *t* statistics in parentheses.

The direct effect of FER is significantly positive (0.0319), indicating that FER has stricter and more direct control means for environmental governance. It can directly promote the local EDQ. The IER's direct (0.0832) and indirect (0.0655) effects are both statistically positive at 5%, proving that it works with neighbors as partners to advance EDQ. The control of pollutants by IER can effectively reduce the spread of contaminants, thus enabling the province's and neighboring provinces' EDQ. The cross product of FER and IER has a significant positive direct (0.0225) and indirect (0.0182) effect, indicating that the dual ER can coordinately promote EDQ and has a positive spatial spillover effect. Hypothesis 4 is further verified.

The direct effects of TS (0.0416) and TR (0.0343) are both significantly positive, indicating that industrial upgrading can promote the high-quality development of the province's economy. Hypothesis 2 is verified. From the perspective of indirect effects, TS (0.0331) between adjacent areas forms an economic development model with neighbors as partners. The reason is that the local TS can encourage the innovative progress's “spillover effect,” which helps to advance nearby regions' technology and encourages an improvement in the EDQ as a whole. The TR (−0.0630) has formed a beggar-thy-neighbor economic development model. Local governments have improved the TR under the influence of various policies, which has aggravated the spatial selection effect of polluting businesses. The manifestation is that enterprises with lower productivity migrate to neighboring cities because they cannot adapt to stricter legal policies, thus reducing the EDQ level of neighboring towns.

The cross product of FER and TR has significant direct and indirect effect coefficients of 0.0139 and 0.0112, respectively, both of which are positive at the 5% level. It shows that TR and FER between adjacent provinces have a development partnership model with neighbors. FER can promote EDQ through TR. The interaction variables between IER and TS have significantly positive direct (0.0597) and indirect (0.0484) influence estimates. It shows that the TS and IER between adjacent provinces have an economic development model with neighbors as partners. It also shows that IER can promote EDQ through the TS. This further validates Hypothesis 3.

Considering all control variables, economic development, marketization, financial development, and technological innovation have significant positive direct, indirect, and total effects on regional EDQ. The local EDQ will encourage the economic development of adjacent provinces through spillover effects due to the enhancement of the economic development level, the marketization level, the financial development level, and the technological innovation level. Overall, the spatial Durbin model's impact decomposition findings indicate the reliability of the aforementioned empirical findings.

### 4.5. Spatial heterogeneity analysis

The exact effects of ER and industrial upgrading on economic development vary depending on the historical, geographical, and social characteristics of different locations in China. Therefore, a discussion of spatial heterogeneity is required. This study divides the research samples into three groups according to geographical location: east, middle, and west. Table 7 shows the empirical results.

**Table 7:** Spatial effect decomposition by region.

**Variables**	**Spatial effect**	**Eastern region**	**Central region**	**Western region**
lnFER	Direct effect	0.0904^***^	0.0231^**^	−0.0115^*^
		(7.25)	(2.36)	(−1.70)
	Indirect effect	0.177^***^	0.0605^**^	0.00117
		(4.82)	(2.14)	(0.70)
	Total effect	0.267^***^	0.0835^**^	−0.0104^*^
		(5.97)	(2.22)	(−1.67)
lnNER	Direct effect	0.201^***^	0.105	−0.124
		(5.62)	(1.50)	(−1.64)
	Indirect effect	0.0840^***^	0.260	0.0109
		(3.60)	(1.50)	(0.60)
	Total effect	0.285^***^	0.365	−0.113
		(5.62)	(1.51)	(−1.55)
lnTS	Direct effect	0.471^***^	−0.0531	−0.00913
		(11.53)	(−1.29)	(−0.38)
	Indirect effect	0.201^***^	−0.0624	0.179^**^
		(3.36)	(−0.36)	(2.39)
	Total effect	0.671^***^	−0.116	0.170^**^
		(7.34)	(−0.56)	(2.14)
lnTR	Direct effect	0.131^***^	0.123^***^	0.0455^***^
		(5.81)	(3.88)	(3.23)
	Indirect effect	0.0546^***^	0.314^*^	0.110^***^
		(3.82)	(1.85)	(3.20)
	Total effect	0.185^***^	0.437^**^	0.155^***^
		(5.95)	(2.21)	(3.87)

^***^, ^**^, ^*^represent the 1, 5, and 10% levels of significance, respectively. *t* statistics in parentheses.

ER and industrial modernization in the three major regions have diverse effects on EDQ from a spatial standpoint. Table 7 shows that FER has both positive direct and indirect effects on the EDQ of the eastern and central areas. The direct effect of ER on the western region is negative, and the indirect effect is not significant. The eastern region's established economy, vibrant market, and comparatively thorough policy framework are the primary causes of this outcome. As manufacturing costs rise due to stricter ER, most businesses can devote more funds to technology advancements that support EDQ in their local and neighboring areas. The center region is less developed than the eastern region, and ER has less impact on fostering the EDQ of the local and surrounding areas. The western region has the lowest degree of development. Local governments prioritize economic development but frequently disregard environmental protection. Implementing ER raises production costs for businesses and impedes regional economic growth.

Both direct and indirect effects of IER on EDQ in the eastern region are significantly positive, which is consistent with the main empirical evidence of this study. IER's direct and indirect effects on EDQ are insignificant in the central and western regions. This conclusion reflects the strong environmental awareness of the public in the eastern region. The population is still largely passive and pays less attention to environmental protection in the central and western regions. FER serves as the basic foundation for improving EDQ. This is related to China's ineffective environmental education and government-led environmental protection system. Industrial structure upgrading has both large positive direct and indirect effects on the eastern region but with no visibly direct effects on the middle and western regions. This condition can be connected to the eastern region's stronger industrial structure. While the indirect impact on the economic growth of the center region is negligible, it has a significantly positive impact on the western region. This demonstrates that improving industrial structure has a considerable favorable spillover effect on the western region. Industrial structure rationalization has a significantly positive direct impact on the EDQ of the eastern, central, and western areas. The direct promotion effect is also greatest in the eastern region, followed by the middle region, and least in the western region. This is related to the degree of structural and industrial development in each region. The indirect effects of industrial structure rationalization are all positive, indicating that industrial structure rationalization has a positive spillover effect on the level of EDQ across the country. Rationalizing the industrial structure between adjacent cities forms an economic development model with neighbors as partners.

## 5. Robustness and endogeneity test

### 5.1. Robustness test

The above empirical results have well verified the theoretical hypothesis of this study. This study employed a geographical distance weight matrix to retest the aforementioned empirical findings and further confirm that the relationship between dual ER, industry transformation and upgrading, and EDQ is solid. The model construction and estimating procedures follow precedents. The results are shown in Table 8. The regression results show that the sign and significance of the explanatory variables in model 1, model 2, and model 3 are consistent with the original model. It shows that the impact of dual ER and industrial transformation and upgrading on EDQ is robust.

**Table 8:** Robustness test.

**Variables**	**Model 1**		**Model 2**		**Model 3**	
	**Direct**	**Indirect**	**Direct**	**Indirect**	**Direct**	**Indirect**
lnFER	−0.0117^**^	−0.0137^**^			0.0284	−0.0257
	(−2.55)	(−2.52)			(1.54)	(−1.31)
lnIER			0.145^***^	0.153^***^	0.0745^*^	−0.116
			(4.01)	(4.35)	(1.68)	(−1.05)
lnFIER					0.0197^**^	0.0165^*^
					(2.01)	(1.86)
lnTS	0.0666^***^	0.0784^***^	0.0483^***^	0.0517^**^	0.0587^***^	0.0498^***^
	(4.02)	(4.05)	(2.72)	(2.57)	(2.98)	(2.61)
lnTR	0.0248^***^	0.0293^**^	0.0365^***^	0.0392^***^	0.0288^***^	0.0242^***^
	(2.61)	(2.52)	(3.73)	(3.28)	(2.96)	(2.64)
lnFERTS	−0.0121	0.101^***^			−0.0308^***^	−0.0262^***^
	(−1.41)	(3.00)			(−3.65)	(−2.91)
lnFERTR	0.00895^*^	−0.0233			0.0144^**^	0.0122^**^
	(1.91)	(−1.27)			(2.30)	(2.11)
lnIERTS			0.0440^*^	0.0474^*^	0.0399^*^	0.0335
			(1.92)	(1.82)	(1.70)	(1.61)
lnIERTR			−0.0604^***^	−0.0651^***^	−0.0646^***^	−0.0545^***^
			(−4.47)	(−3.59)	(−4.45)	(−3.58)
lnVgdp	0.104^***^	0.122^***^	0.0560^***^	0.0594^***^	0.123^***^	0.103^***^
	(7.80)	(7.66)	(5.38)	(5.70)	(8.21)	(5.35)
lnVmark	0.122^***^	0.145^***^	0.236^***^	−0.145^***^	0.141^***^	0.119^***^
	(6.32)	(5.37)	(8.07)	(−2.73)	(7.65)	(5.06)
lnVfin2	0.130^***^	0.153^***^	0.0944^***^	0.101^***^	0.120^***^	0.127^*^
	(4.85)	(4.66)	(3.74)	(3.55)	(4.06)	(1.79)
lnVrd	0.0303^**^	−0.000211	0.0564^***^	0.0604^***^	0.0429^***^	0.0360^***^
	(2.04)	(−0.00)	(3.95)	(3.55)	(3.07)	(2.88)

^***^, ^**^, ^*^represent the 1, 5, and 10% levels of significance, respectively. *t* statistics in parentheses.

### 5.2. Endogeneity test

The increase in the level of EDQ may also have an impact on local ER policies. To avoid the resulting error caused by endogeneity in the regression, the GMM system is used to re-estimate the original equation. System GMM is a commonly used method for endogenous testing. A consistent estimate can be generated by including the lagged items of endogenous explanatory variables as instrumental variables in the regression equation. The estimated results are shown in Table 9. The regression findings show that all of the *p*-values for the AR (1) test are below 0.01, whereas all of the *p*-values for the AR (2) test are over 0.01. The residual term exhibits first-order autocorrelation, whereas second-order autocorrelation is absent. The regression results passed the serial correlation test. All of the *p*-values for the Hansen test were higher than 0.1, demonstrating the validity and rationale of the instrumental variables. ER has an inhibitory effect on EDQ, and NER has a promoting effect. The synergy between the two can significantly improve EDQ. The upgrading of the industrial structure can promote the regional EDQ, which is the same as the result of panel regression.

**Table 9:** Endogeneity test.

**Variables**	**Model 1**	**Model 2**	**Model 3**
L.lnEDQ	0.569^***^	0.640^***^	0.573^***^
	(18.16)	(23.66)	(18.28)
lnFER	−0.018^***^		0.009
	(−5.59)		(0.58)
lnIER		0.004	−0.033
		(0.38)	(−1.63)
lnFIER			0.016^**^
			(2.06)
lnTS	0.040^***^	0.043^***^	0.034^**^
	(3.12)	(3.27)	(2.68)
lnTR	0.012^*^	0.020^**^	0.011^*^
	(1.80)	(2.57)	(1.74)
lnFERTS	−0.000		0.001
	(−0.07)		(0.17)
lnFERTR	0.004		−0.002
	(1.02)		(−0.36)
lnIERTS		0.005	0.005
		(0.21)	(0.19)
lnIERTR		−0.012	−0.013
		(−1.36)	(−1.39)
lnVgdp	0.122^***^	0.092^***^	0.121^***^
	(12.63)	(10.94)	(11.96)
lnVmark	0.082^***^	0.047^**^	0.093^***^
	(4.15)	(2.71)	(4.56)
lnVfin2	0.120^***^	0.113^***^	0.128^***^
	(6.91)	(5.95)	(7.05)
lnVrd	0.039^***^	0.027^***^	0.038^***^
	(5.01)	(4.07)	(5.08)
Constant	−0.827^***^	−0.706^***^	−0.904^***^
	(−11.15)	(−7.98)	(−9.60)
AR(1)	0.000	0.000	0.000
AR(2)	0.171	0.169	0.189
Hansen	0.745	0.718	0.796

^***^, ^**^, ^*^represent the 1, 5, and 10% levels of significance, respectively. *t* statistics in parentheses.

## 6. Conclusions and policy implications

This study built a spatial Durbin model to investigate the effects of dual ER and industry upgrading on EDQ based on theoretical analysis. The following are the primary conclusions: (1) The EDQ has visible spatial heterogeneity, and regions show strong local spatial agglomeration characteristics. (2) The impact of ER on EDQ is heterogeneous. FER has a restraining effect, while IER has a promoting effect. Dual ER interacts to promote EDQ. (3) The TS has a direct role in promoting EDQ and forms an economic development model of neighbors as partners; the TR has a direct role in promoting EDQ and forms an economic development model of beggar-thy-neighbor. (4) The TR is the main channel for FER to promote EDQ, and the TS is the main channel for IER to promote EDQ. (5) The impact of ER on EDQ is spatially heterogeneous and plays a greater role in developed regions.

This study provides the following policy recommendations based on the aforementioned findings.

First, the government should implement differentiated action for diverse ER and fully account for the variety of ER when formulating regulatory policies. Policymakers should find a balance between the combination of FER and IER and give full play to the policy effect of ER to promote EDQ. Second, local governments should not blindly introduce enterprises due to industrial transfer caused by ER. Still, they should establish appropriate industrial support development mechanisms to maximize the synchronization and complementarity among constructions in various locations and the judicious distribution of resources. Additionally, it is crucial to strengthen cooperation between regions, prevent the formation of the pollution shelter effect, and encourage the coordinated development of the industrial structure. Third, the government should formulate corresponding ER policies according to the status quo of different industrial structures. FER should be the primary focus when the industrial system is dominated by sectors with high energy requirements and emissions. The government should now create stringent regulatory measures, plan and guide enterprises toward clean production, and encourage regional industrial structure upgrades. IER should be the main focus in areas dominated by knowledge- and technology-intensive industries. The government should gradually adjust the environmental protection policy, stimulate the innovative compensation effect of ER, implement the regional coordinated development strategy, speed up the establishment of a new growth pattern, and effectively push regional coordinated development.

Future work will resolve a few of this study's limitations. Scholars have defined the selection of indicators from different perspectives. The metrics chosen within that work were derived from existing research. The innovation and comprehensiveness of indicators need to be further improved. This study mainly analyzes the implementation effect of environmental policies and the direction of industrial transformation and upgrading at the provincial level. It is necessary to further study the prefecture-level city level and deeply analyze the implementation path of regionally coordinated development.

## Data availability statement

The datasets presented in this study can be found in online repositories. The names of the repository/repositories and accession number(s) can be found below: China Statistical Yearbook (http://www.stats.gov.cn/tjsj/ndsj/, accessed on 6 June 2022), China Environmental Yearbook (http://www.stats.gov.cn/ztjc/ztsj/hjtjzl/, accessed on 6 June 2022), and the EPS database (http://www.epsnet.com.cn/, accessed on 6 June 2022).

## Author contributions

YL: conceptualization, software, resources, and data curation. WL: validation and supervision. YL and WL: methodology, formal analysis, writing—original draft preparation, and writing—review and editing. All authors have read and agreed to the published version of the manuscript.
